# Safety and Efficacy of Oral Mifepristone for Cervical Ripening and Induction of Labor

**DOI:** 10.7759/cureus.65450

**Published:** 2024-07-26

**Authors:** Pratap Pharande, Ananya R Kiran, Shivani Patel, Hada V Vanrajsinh

**Affiliations:** 1 Obstetrics and Gynecology, Dr. D. Y. Patil Medical College, Hospital and Research Center, Dr. D. Y. Patil Vidyapeeth, Pune (Deemed to be University), Pune, IND

**Keywords:** dilatation of cervix, uterine contraction, bishop score, mifepristone, labor augmentation

## Abstract

Background

Labor induction, a common practice to prevent maternal and fetal complications from prolonged labor, involves stimulating contractions before they begin naturally. This can be achieved through medications, mechanical methods, or surgical interventions. Cervical ripening is crucial for successful delivery. When the cervix is not sufficiently ripe, drugs are often used to augment this process chemically.

Objective

To evaluate the safety and efficacy of mifepristone for cervical ripening and induction of labor.

Method

A sample size of 200 was used in this single-blind randomized control trial. Primarily, pregnant women with term pregnancies, Bishop scores <6, and cephalic fetal presentation were included in the study. The study population was randomly divided into test and control groups. The test group (n=100) was administered 200 mg of mifepristone orally, while the control group (n=100) received a placebo. The Bishop score was reassessed 24 hours after mifepristone administration. Patients were taken for labor induction if their Bishop score was >6. For individuals with a Bishop score of <6, 1 mg of dinoprostone gel was administered intracervically once every six hours. Safety and efficacy were assessed by analyzing several parameters associated with labor progression, maternal outcomes, and fetal outcomes.

Results

The mean age of patients in the test group was 26±4.5 years, while in the control group, it was 26±5 years. The induction-to-delivery interval was notably shorter in the test group (18.8±2.3 hours) than in the control group (19.24±1.8 hours, p<0.0001). After the administration of 200 mg mifepristone, the mean Bishop score in the test group was 5.74±0.8, compared to 5.13±0.76 in the control group. The increase in the Bishop score after mifepristone treatment was significantly higher in the test group than in the control group (p-value=0.013). In the study, 73 (73%) patients in the test group had a normal vaginal delivery (NVD), whereas NVD accounted for 64 (64%) patients in the control group. Instrumental deliveries were less frequent in the test group, accounting for 14 (14%) patients, compared to 16 (16%) patients in the control group. The frequency of lower segment cesarean section (LSCS) was also lower in the mifepristone-treated group at 13 (13%) compared to the control group at 20 (20%). Fetal distress in five (38%) patients and non-progression of labor in 11 (55%) patients were the most frequent indications for LSCS in the test and control groups, respectively. There was no significant difference in neonatal outcomes between the test and control groups. Meconium-stained liquor was the most frequent complication in both the test group (10, 10%) and the control group (5, or 5%).

Conclusion

Administration of mifepristone effectively increased the Bishop scores and reduced the induction-to-delivery interval compared to controls, highlighting its potential as a cervical ripening agent.

## Introduction

Labor is defined as the beginning of effective uterine contractions that lead to the gradual effacement and dilation of the cervix [1]. Favorable anatomical features of the reproductive system, including cervical ripening and stimulation of uterine contractions, enable spontaneous labor and delivery. A myriad of signaling pathways allow progressive changes in the cervix and prime it for delivery. One of the major pathways involved is the inflammatory signaling pathway, which is upregulated and leads to an increase in the synthesis of prostaglandins. Prostaglandins function to antagonize progesterone signaling and activate metalloproteases that disintegrate collagen fibers in the cervical stroma [2]. The progressive inputs from these signaling pathways result in cervical dilation and effacement, changing the cervix's consistency from firm to “paper thin,” thereby paving the way for normal delivery. The Bishop score is used to assess cervical status before induction. The Bishop score is an assessment tool used to evaluate the condition of the cervix for labor by measuring the parameters encompassing cervical dilation, position, effacement, consistency of the cervix, and fetal station. Each parameter is scored, and the total score helps to predict the likelihood of spontaneous labor and the potential need for induction [3].

In cases where delivery cannot be postponed and the cervix is not sufficiently prepared for delivery, exogenous intervention is required to prevent perinatal and maternal complications [4,5]. There are also several indications for labor induction based on the medical and obstetric history of the patient. Placental and fetal indications include chronic placental insufficiency, multiple gestations, growth restriction, premature rupture of membranes, chorioamnionitis, oligohydramnios or polyhydramnios, alloimmunization, and preterm prelabor rupture of membranes (PPROM). Maternal medical indications for induction encompass hypertensive disorders, renal disease complicating pregnancy, and pre-gestational and gestational diabetes mellitus (GDM) [6,7]. Conversely, contraindications for labor induction include placenta accreta spectrum, transverse fetal presentation, umbilical cord prolapse, cervical carcinoma, active herpes infection, and previous myomectomy breaching the endometrial cavity [6,7].

Augmentation of labor is required in cases of inadequate spontaneous contractions to enhance cervical dilatation and cause fetal descent. Several mechanical, surgical, and chemical methods of expediting cervical ripening and labor have been practiced safely in the past [8]. In this study, we will focus solely on chemical/medicinal methods. Among chemical methods, prostaglandins E1 (PGE1) (misoprostol) and E2 (PGE2) (dinoprostone) have been mostly used in the past for the induction of labor. However, only PGE2 is currently licensed for this purpose. Mifepristone (RU-486), a 19-non-steroid progesterone antagonist previously known for its role in the termination of pregnancy during the early first and second trimesters, has recently gained attention for its use in the ripening of the cervix and induction of labor at term [9]. Mifepristone acts as an antagonist to progesterone and has a higher affinity for progesterone receptors than progesterone itself. Pharmacokinetic studies of mifepristone show that it is quickly absorbed and has a relatively longer half-life (25-30 hours). The key metabolites also have a higher affinity for progesterone receptors [10]. Studies have shown that mifepristone is effective for inducing labor and improving cervical readiness, as it significantly increases the likelihood of being in labor or having a favorable cervix within 48 and 96 hours compared to a placebo. It reduces the need for oxytocin augmentation and decreases the likelihood of cesarean sections, particularly due to failure to induce labor [11].

## Materials and methods

Study design

This single-blind randomized control trial included a sample size of 200 pregnant women with term pregnancies and Bishop scores <6, with cephalic fetal presentation. After obtaining detailed medical histories, performing examinations, confirming diagnoses, conducting necessary investigations, and obtaining informed consent, the participants were randomly assigned to either the test group or the control group.

Sample size

The sample size of 200 participants (100 per group) was determined to be sufficient to detect a clinically meaningful difference in primary outcomes with 80% power and a significance level of 0.05. This ensures the reliability and validity of the study results.

Group allocation and intervention

The participants were randomly divided into two groups: the test group (n=100) and the control group (n=100). The test group received 200 mg of mifepristone orally, while the control group was given a placebo. The Bishop score was reassessed 24 hours after administration. Patients with a Bishop score >6 were induced for labor. For those with a Bishop score <6, 1 mg of dinoprostone gel was administered intracervically every six hours until labor induction was achieved.

Inclusion criteria

The inclusion criteria for this study encompassed live singleton pregnancies with cephalic fetal presentation. This included term pregnancies that were post-dated, affected by preeclampsia, or complicated by GDM. Additionally, pregnancies with an unripe cervix, defined by a Bishop score <6, and intact membranes at the time of admission, were included. These criteria aimed to focus on pregnancies with specific characteristics that might benefit from the intervention being studied, such as labor induction.

Exclusion criteria

The exclusion criteria included pregnancies with premature rupture of membranes, chorioamnionitis, or cephalopelvic disproportion. Pregnancies with a history of previous lower segment cesarean section (LSCS) or multiple gestations were also excluded. Additionally, pregnancies with abnormal fetal heart rate patterns were excluded from the study.

Data collection and analysis

Safety and efficacy were evaluated by analyzing several parameters associated with labor progression, maternal outcomes, and fetal outcomes. Data were analyzed using GraphPad Prism 10 (GraphPad Software, San Diego, CA, USA). Continuous variables were expressed as means and percentages. Bar graphs with standard error of the mean (SEM) were used for visual representation. Paired Student's t-test and Wilcoxon test were employed to compare differences between the test and control groups, with a p-value of less than 0.05 considered statistically significant.

## Results

In our study, the mean age of patients in the test group was 26±4.5 years, while the mean age of patients in the control group was 26±5 years. There was no difference in the mean gestational age between the test group and the control group. The mean gestational age in both groups was 38 weeks (Table 1).

**Table 1:** Demographic details

	Test group	Control group
Mean age (years)	26±4.5	26±5
Mean gestational age (weeks)	39±2	38.98±1.7

**Figure 1:**
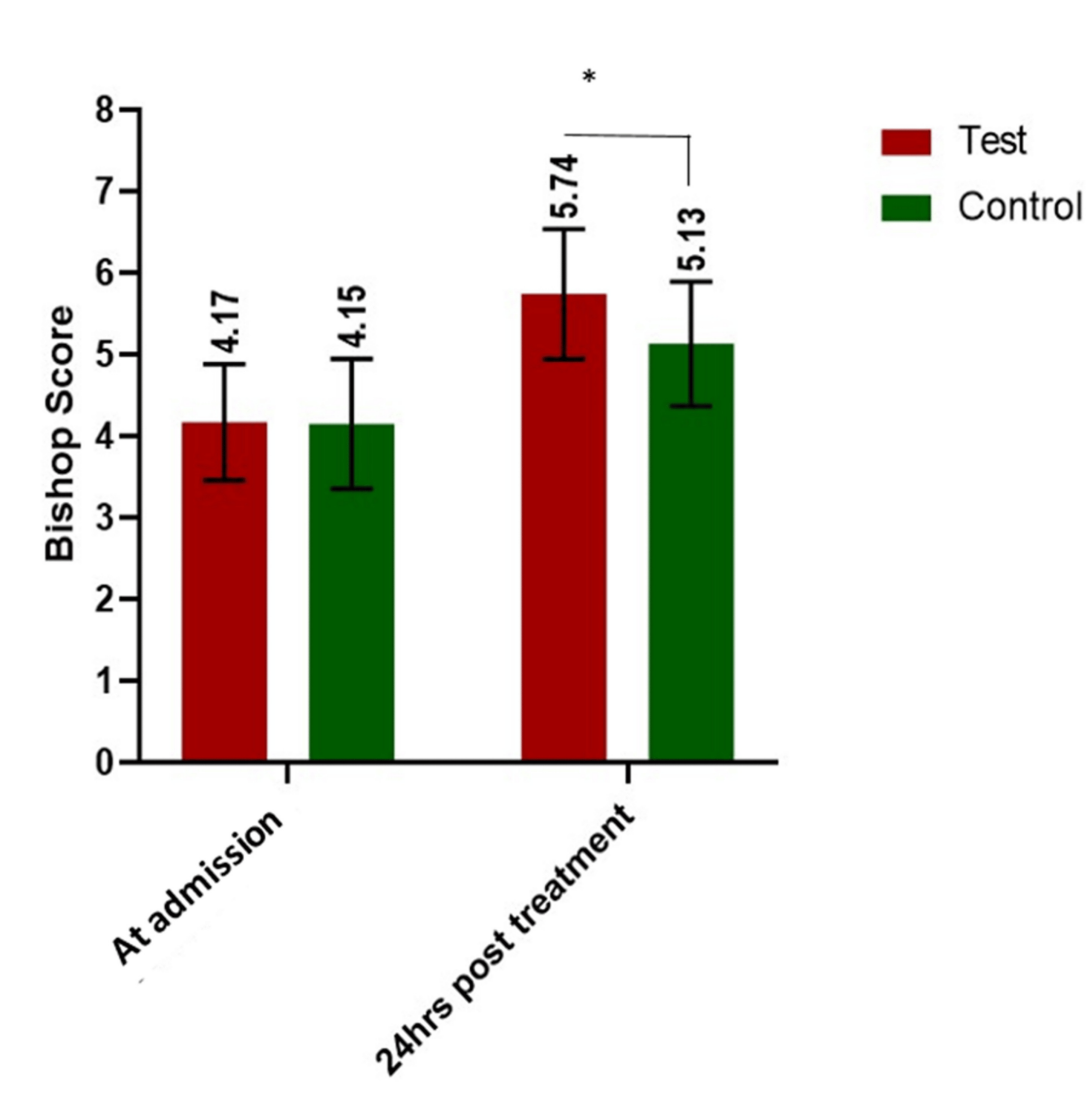
Comparison of Bishop score at the time of admission and 24-hour post-treatment with 200 mg mifepristone (*p value=0.013)

The Bishop score was assessed at the time of admission and reassessed 24 hours after the administration of 200 mg mifepristone (Table 2). The mean Bishop score at the time of admission was 4.17±0.71 in the test group and 4.15±0.8 in the control group; however, the mean Bishop score after the administration of mifepristone in the test group was found to be 5.74±0.8 and 5.13±0.76 in the control group (Figure 1). Although the Bishop score increased in both the test and control groups, the increase in the Bishop score after mifepristone treatment was significantly higher in the test group when compared to the increase in the control group (p-value=0.013). The mean induction-to-delivery interval was 18.8±2.3 hours in the test group and 19.24±1.8 hours in the control group (p<0.0001) (Figure 1).

**Table 2:** Bishop score at the time of admission and 24-hour post-treatment with 200 mg mifepristone

	Test group	Control group
Mean Bishop score at admission	4.13±0.73	4.15±0.8
Mean Bishop score at 24 hours post-treatment	5.95±1	5.65±0.74
Mean induction to delivery time (hours)	18.8±2.3	19.24±1.8

Several obstetric conditions are critical concerning maternal and fetal outcomes, thereby indicating the need for induction. In our study population, we assessed the indications for induction and found that postdatism was the most frequent indication (61, or 30.5%), followed by pregnancy-induced hypertension (PIH) at 33 (16.5%), GDM at 32 (16%), pre-eclampsia at 29 (14.5%), oligohydramnios at 24 (12%), and intrauterine growth restriction (IUGR) with normal fetal Doppler at 21 (10.5%) (Table 3).

**Table 3:** Distribution of indication for induction PIH: Pregnancy-induced hypertension; GDM: Gestational diabetes mellitus; IUGR: Intrauterine growth restriction

Indication for induction	Test group		Control group		Total	
	Frequency	Percentage	Frequency	Percentage	Frequency	Percentage
Postdatism	28	28	33	33	61	30.5
PIH	15	15	18	18	33	16.5
Oligohydramnios	13	13	11	11	24	12
GDM	15	15	17	17	32	16
Pre-eclampsia	17	17	12	12	29	14.5
IUGR	12	12	9	9	21	10.5

As previously mentioned the Bishop score was reassessed 24 hours after the tablet mifepristone was administered orally. In cases where the Bishop score was <6, dinoprostone gel (0.5 mg single dose) was given intracervical six hours apart in case of multiple doses. In our study population, single dose of dinoprostone was administered to 80 (40%) patients with 33 (33%) and 47 (47%) patients in test and control groups, respectively. A total of 35 (17.5%) patients in the study population received two doses with the test and control group receiving 14 (14%) and 21 (21%) respectively. Three doses were given to 22 (11%) patients in the total population with the test and control group accounting for 12 (12%) and 10 (10%), respectively (Table 4).

**Table 4:** Distribution based on dinoprostone doses administered

Dinopristone (0.5 mg) gels used	Test group		Control group		Total	
	Frequency	Percentage	Frequency	Percentage	Frequency	Percentage
One	33	33	47	47	80	40
Two	14	14	21	21	35	17.5
Three	12	12	10	10	22	11

In our study population, 73 (73%) patients in the test group had a normal vaginal delivery (NVD), while in the control group, NVD was less and accounted for 64 (64%) of the population. Instrumental deliveries were less frequent in the test group, accounting for 14 (14%) patients, compared to the control group, which included 16 (16%) patients. Interestingly, the frequency of LSCS was also less in the mifepristone-treated group, accounting for 13 (13%) compared to the control group, which accounted for 20 (20%) (Table 5).

**Table 5:** Distribution based on mode of delivery NVD: Normal vaginal delivery; LSCS: Lower segment cesarean section

Mode of delivery	Test group		Control group	
	Frequency	Percentage	Frequency	Percentage
NVD	73	73	64	64
LSCS	13	13	20	20
Instrumental	14	14	16	16
Total	100	100	100	100

Several factors were evaluated when determining the need for an LSCS. The most frequent indication in the test group was fetal distress (5, or 38%); however, it accounted for only seven (35%) of patients in the control group. Nonprogression of labor (NPOL) was the most frequent indication in the control group and accounted for 11 (55%) patients. Interestingly, it only accounted for five (38%) patients in the test group. Other indications included failed induction (test group: 4, or 31%; control group: 0, or 0%) and abruption (test group: 0, or 0%; control group: 2, or 10%) (Table 6).

**Table 6:** Distribution based on indication for induction NPOL: Nonprogression of labor; LSCS: Lower segment cesarean section

Indication for LSCS	Test group		Control group	
	Frequency (N=13)	Percentage	Frequency (N=20)	Percentage
Failed induction	4	31	0	0
Fetal distress	5	38	7	35
NPOL	4	31	11	55
Abruptio placenta	0	0	2	10
Total	13	100	20	100

There was no difference in the neonatal outcome in both the test and control groups. In the test group, 18 (18%) newborns were shifted to the neonatal intensive care unit (NICU), while 17 (17%) newborns in the control group were admitted to the NICU (Table 7).

**Table 7:** Distribution based on neonatal outcomes NICU: Neonatal intensive care unit

NICU	Test group		Control group	
	Frequency	Percentage	Frequency	Percentage
Yes	18	18	17	17
No	82	82	83	83
Total	100	100	100	100

To determine the safety of mifepristone, we assessed the associated complications. We found that 10 (10%) mifepristone-treated patients had meconium-stained liquor followed by uterine hypercontraction and uterine tachysystole in eight (8%) and five (5%) patients in the test group, respectively. Similar complications were found in the control group, however at a lower frequency. Meconium-stained liquor was reported in five (5%) patients followed by uterine tachysystole in two (2%) patients in the control group. There was no case of uterine hypercontraction in the control group (Table 8).

**Table 8:** Distribution based on maternal complications

Complications	Test group		Control group	
	Frequency	Percentage	Frequency	Percentage
Meconium stained liquor	10	10	5	5
Uterine tachysystole	5	5	2	2
Uterine hypercontraction	8	8	0	0

## Discussion

Augmentation of labor is required in cases of inadequate spontaneous contractions to enhance cervical dilatation and cause fetal descent. As discussed previously, inhibition of the progesterone signaling pathway is critical for cervical effacement. However, in certain cases where the cervix is unfavorable for delivery, chemical methods are used to enhance the cervical effacement process. Mifepristone (RU-486), a 19-nonsteroid drug, has been widely studied for its role in cervical ripening and induction. Mifepristone has a strong affinity for progesterone receptors and acts as an antagonist by inhibiting the progesterone downstream signaling cascade. Additionally, it sensitizes the uterus to prostaglandins, causing cervical dilation, which, in turn, induces labor.

In a prospective study comparing the efficacy of mifepristone, Mandade and Bangal (2016) demonstrated a significant increase in the Bishop score following mifepristone treatment [12]. Another study by Mohapatra and Samarpita (2020) reported that 90% of mifepristone-treated patients showed an increase in the Bishop score after six hours [13]. These findings are consistent with our study, as we also observed a significant increase in the mean Bishop score in the mifepristone treatment group compared to the placebo group. Furthermore, our study revealed a significant decrease in the mean induction-to-delivery interval (p<0.0001). Several studies, including those by Giacalone (1998), Wing (2000), Wing et al. (2005), Baev et al. (2017), and Edwards (1996), have also reported a significant reduction in the induction-to-delivery interval following mifepristone treatment [14-17].

In this study, we observed a slight decrease in the frequency of deliveries through LSCS after mifepristone treatment. Several reports support this finding. Edwards (1996), Wing (2000), and Hapangama and Neilson (2009) have shown that women treated with mifepristone are less likely to undergo LSCS during delivery [14,17,18]. However, Baev et al. (2017) reported no significant difference in the mode of delivery between the mifepristone-treated group (n=74) and the control group (n=74) [16]. We also found that fetal distress was the most frequent indication for LSCS in the mifepristone-treated group. Elliot (1998) and Wing et al. (2005) similarly reported fetal distress as the most common reason for LSCS [15,19].

Mifepristone and its metabolites have been shown to diffuse across the placenta, necessitating a thorough evaluation of its effects on the fetus and neonate [20]. In our study, there was no significant difference in neonatal outcomes, with 18 (18%) neonates in the test group and 17 (17%) neonates in the control group being admitted to the NICU. Previous studies, including Wing (2000), Mandade and Bangal (2016), Baev et al. (2017), and Hapangama and Neilson (2009), also report no difference in neonatal outcomes between the mifepristone-treated group and the placebo group [11,14,17,18].

We finally evaluated maternal complications to assess the safety of mifepristone. We found that 10 (10%) patients in the mifepristone-treated group had meconium-stained liquor, followed by uterine hypercontraction and uterine tachysystole in eight (8%) and five (5%) patients in the test group, respectively. Similar complications were found in the control group, though at a lower frequency: meconium-stained liquor was reported in five (5%) patients, followed by uterine tachysystole in two (2%) patients of the control group. There were no cases of uterine hypercontraction in the control group. Consistent with our findings, Wing et al. (2005) reported that 9% of mifepristone-treated patients had meconium-stained liquor [15].

This study's limitations include its sample size, which may not have provided sufficient power to detect smaller yet clinically relevant differences in maternal complications. The single-blind design introduces potential observer bias, while the exclusion of certain populations limits the generalizability of the findings. Additionally, the study's focus on short-term outcomes and variability in dinoprostone dosing might affect result consistency. Conducted at a single center, the study's findings may not be applicable to broader or more diverse populations.

## Conclusions

We conclude that administering a single dose of 200 mg mifepristone significantly improves the Bishop score and reduces the induction-to-delivery interval. Our findings align with previous studies, demonstrating that mifepristone effectively enhances cervical ripening and accelerates labor while presenting no major adverse maternal or neonatal complications. This highlights mifepristone's potential as an effective agent for cervical ripening in labor induction protocols.
